# Low Control over Palatable Food Intake in Rats Is Associated with Habitual Behavior and Relapse Vulnerability: Individual Differences

**DOI:** 10.1371/journal.pone.0074645

**Published:** 2013-09-10

**Authors:** Johannes W. de Jong, Karin E. Meijboom, Louk J. M. J. Vanderschuren, Roger A. H. Adan

**Affiliations:** 1 Rudolf Magnus Institute of Neuroscience, Department of Neuroscience and Pharmacology, University Medical Center Utrecht, Utrecht, The Netherlands; 2 Department of Animals in Science and Society, Division of Behavioural Neuroscience, Faculty of Veterinary Medicine, Utrecht University, Utrecht, The Netherlands; Sapienza University of Rome, Italy

## Abstract

The worldwide obesity epidemic poses an enormous and growing threat to public health. However, the neurobehavioral mechanisms of overeating and obesity are incompletely understood. It has been proposed that addiction-like processes may underlie certain forms of obesity, in particular those associated with binge eating disorder. To investigate the role of addiction-like processes in obesity, we adapted a model of cocaine addiction-like behavior in rats responding for highly palatable food. Here, we tested whether rats responding for highly palatable chocolate Ensure would come to show three criteria of addiction-like behavior, i.e., high motivation, continued seeking despite signaled non-availability and persistence of seeking despite aversive consequences. We also investigated whether exposure to a binge model (a diet consisting of alternating periods of limited food access and access to highly palatable food), promotes the appearance of food addiction-like behavior. Our data show substantial individual differences in control over palatable food seeking and taking, but no distinct subgroup of animals showing addiction-like behavior could be identified. Instead, we observed a wide range extending from low to very high control over palatable food intake. Exposure to the binge model did not affect control over palatable food seeking and taking, however. Animals that showed low control over palatable food intake (i.e., scored high on the three criteria for addiction-like behavior) were less sensitive to devaluation of the food reward and more prone to food-induced reinstatement of extinguished responding, indicating that control over palatable food intake is associated with habitual food intake and vulnerability to relapse. In conclusion, we present an animal model to assess control over food seeking and taking. Since diminished control over food intake is a major factor in the development of obesity, understanding its behavioral and neural underpinnings may facilitate improved management of the obesity epidemic.

## Introduction

Obesity is a major threat to public health, because it increases the risk for diabetes, cardiovascular disease and cancer [1,2]. Prevalence rates of obesity have been steadily increasing with an expected increase by 2030 of 65 million and 11 million obese adults in the USA and UK, respectively [2]. The current prevalence of obesity (defined as a body mass index > 30 kg/m^2^) is about 33% in the US and more than half of the member states of the EU have obesity levels >20% [3,4]. Despite its high prevalence, the neural and behavioral underpinnings of obesity are incompletely understood.

It has been suggested that certain forms of excessive food intake associated with obesity are mediated by an addiction-like process [1,2,5–11]. Although the extent to which food addiction could explain the obesity epidemic is subject to intense debate [2,12–14]. In support of a role of addiction-like processes in obesity, there is overlap between the DSM-IV criteria for substance dependence and the proposed criteria for binge eating disorder [3,4,15,16] and obesity [1,2,17]. Furthermore, the comorbidity between eating disorders and substance abuse disorders may be as high as 40% [18]. In this respect it has been suggested that (over) eating and drug use rely on similar neural circuitry [19]. One possible shared neural mechanism is a decrease in dopamine D2 receptor availability in the striatum that is found in both disorders [20–24], a finding that was confirmed in an animal model of compulsive eating [25]. Other similarities include a similar brain activity pattern following craving and suppression of craving [26–30] and co-occurrence with an impulsive personality or Attention Deficit Hyperactivity Disorder [31–36].

We have previously argued that recently developed models from the drug addiction field may be useful to investigate the concept of food addiction [15]. In 2004, Deroche-Gamonet et al. developed a model for addiction-like behavior in rats, based on loss of control over cocaine intake [37]. In this model, rats self-administered cocaine daily for several months. The animals were tested for three behavioral parameters based on the DSM-IV criteria for substance dependence, i.e. 1) Difficulty limiting seeking during signaled non-availability. 2) Extremely high motivation to seek and take the drug. 3) Continued seeking of the drug despite aversive consequences. It was found that a subgroup of rats (17,2%) scored within the upper tertile for each criterion, which is far more than would be expected by chance (i.e., 3,6%). In addition, these addiction-like behavior-expressing animals appeared to be more vulnerable to reinstatement of extinguished drug seeking, a model for relapse to drug abuse after detoxification [38].

In the present study, we tested whether addictive behavior directed at food can be demonstrated using a similar approach as Deroche-Gamonet et al. In order to facilitate the appearance of food addiction-like behavior we exposed animals to a binge-model consisting of alternating periods of food restriction and access to palatable food. Binge eating models consisting of either intermediate access to palatable food [39,40] or alternating (12h/12h) access to sucrose and food deprivation have been shown to mediate bingeing [41] and certain aspects of addiction such as withdrawal symptoms [42,43] as well as changes in dopamine signaling that are also seen after prolonged drug exposure [44,45].

It has been proposed that the development of addiction is facilitated by a switch from outcome-driven, goal-directed behavior to a habitual, stimulus-response structure of behavior [46,47]. In order to test the role of habitual behavior in our proposed model of food addiction-like behavior, we also tested responding for food after devaluation of the palatable food reinforcer [48]. Moreover, since addiction-like behavior is associated with increased vulnerability to reinstatement of drug seeking [37], we hypothesized that animals with less control over their food intake would be more prone to cue and food-induced reinstatement of food seeking after extinction.

## Materials and Methods

### Ethics Statement

Experiments were approved by the Animal Ethics Committee of Utrecht University and were conducted in agreement with Dutch laws (Wet op de Dierproeven, 1996) and European regulations (Guideline 86/609/EEC).

### Animals

6 week old male Wistar rats (Charles River, Sulzfeld, Germany) weighing 150-200 grams at the beginning of experiment were individually housed in Macrolon cages (L = 40 cm, W = 25 cm, H = 18 cm) under controlled conditions (temperature 20–21 °C, 55±15% relative humidity) and under a reversed 12 hour light-dark cycle (lights on at 19.00 h). Chow and water were freely available. All experiments were conducted during the dark phase of the day-night cycle.

### Experimental overview

In adapting the Deroche-Gamonet model for loss of control of cocaine seeking to palatable food seeking, we found in a pilot study that even mild electric footshock suppressed all food seeking. We therefore chose to measure ‘continued seeking despite punishment’ using quinine adulteration of the palatable food [49]. This pilot experiment compared 4 diets (described below) for their potency to evoke food addiction-like behavior. In this case 24 animals (n=6 per group) were trained and tested on the three behaviors as described by [37]. Interestingly, when the animals were tested for the third criterion (resistance to mild electric footshock), a complete suppression of chocolate seeking was found, even when the shock intensity was lowered to 0.35 mA. No difference in responding under the shock paradigm was found between the different diet groups (ANOVA p=0.1146 F=2.243 df = 23). Additionally, we did not observe a significant difference in responding under a progressive ratio schedule of reinforcement between the four diet groups (data not shown). We did, however, observe a trend towards an increase in addiction-like behavior in animals exposed to the binge model when we took all three criteria into consideration. Since electric footshock suppressed all reward seeking, we chose to measure the criterion of resistance to adversity in a different way, i.e. by exposing the animals to the palatable food adulterated with 2 mM quinine. In the main experiment described in the present study, we compared a group exposed to the binge model (n=36) to a chow-fed control group (n=12). For this experiment, the animals were pre-trained on the three criteria for 5 weeks followed by 8 weeks of access to the diet. We did not observe a difference in operant responding between the diet groups before the diet. We then continued by retraining and testing on the three criteria followed by 10 extinction sessions and two reinstatement (cue- and chocolate induced) sessions.

### Diets

Four different diets were used in this study, and animals were exposed to the respective diets for 8 weeks. The control diet consisted of ad libitum chow (SDS, 3.3 kcal/g, 77.0% carbohydrate, 2.8% fat, 17.3% protein). The restricted access diet consisted of ad libitum chow supplemented with 3h access to chocolate Ensure^TM^ (Abbott Laboratories, Abbott Park, IL, USA), for 5 days a week (from 12.00–15.00h). The high-fat high-sucrose choice diet consisted of ad libitum chow in combination with ad libitum saturated fat (Beef tallow (Ossewit/Blanc de Boeuf), Vandemoortele, Belgium, 9.1 kcal/g) and a 30% sucrose solution (commercial grade sucrose in tap water, 1.2 kcal/ml). The binge diet consisted of 4 days of 15.0-15.5g chow/day alternated with 3 days of ad libitum chow supplemented with ad libitum Oreo cookies (Nabisco, East Hanover, NJ, USA, 4.7 kcal/g, 74% carbohydrates, 21% fat, 3% protein). In this case the Oreo cookies were available for 24h/day for three days. The 15g chow/day was based on previous work by Hagan et al. where animals were restricted to 66% of ad-lib chow. This model is a modified version of Hagan et al. without the stress component of the binge-model [39,50]. Tap water was available at all times, except during testing. A pilot study compared all four diets. Animals were tested before and after 8 weeks of access to the diets. The main experiment of this article compares 8 weeks of binge diet to 8 weeks of ad-lib chow. We continued with the binge diet because data from literature, as well as our own pilot data suggested that a binge diet as described above is most likely to evoke food addiction-like behavior [39].

### Apparatus

Rats were trained in operant conditioning chambers (30.5 x 24.1 x 21.0 cm; Med Associates Inc, St. Albans, VT, USA). Each chamber was equipped with two retractable levers (4.8 x 1.9 cm). Above each lever a cue light was located (ENV-221M stimulus light for rats, 28V, 100mA; Med Associates Inc) and a house light (ENV-215M house light for rat chambers, 28 V, 100mA; Med Associates Inc) was placed on the opposite wall. The floor of the chamber was covered with a metal grid with bars separated by 1 cm. The chamber was placed in a sound attenuating cubicle equipped with a ventilation fan to minimize external noise. Chocolate Ensure was delivered to a food receptacle, located in between the two levers, via nylon tubing attached to a single speed syringe pump (PHM-100-3.33; Med Associates Inc) placed outside the chamber. The operant chamber was controlled by MED-PC (version IV) Research Control & Data Acquisition System software.

### Acquisition of chocolate Ensure self administration

Animals were trained to respond for food as described before [51,52]. The rats first received 10 operant training sessions lasting 1 h. During these sessions, two levers were present, one of which was designated as active. The position of the active and inactive levers was counterbalanced between animals. A session started with insertion of both levers and illumination of the house light. During the first session, a fixed ratio (FR) 1 schedule of reinforcement was used, meaning that each active lever press resulted in the delivery of 0.2 ml chocolate Ensure, retraction of both levers for 20 sec and illumination of the cue light above the active lever for 10 sec during which the house light was turned off. The response requirement was increased to a FR2 schedule of reinforcement during the second and third session. From the fourth session onwards, a FR5 schedule of reinforcement was enforced.

### Time-out responding

The time-out procedure was based on [37], although a shorter session duration was used to prevent effects of satiety on responding. Sessions consisted of 5 blocks of 10 min chocolate Ensure availability interchanged with 4 blocks of 5 min during which chocolate Ensure was unavailable. During availability blocks, the response-contingent presence of the reward was indicated to the animals by illumination of the house light. The self-administration procedure during availability blocks was the same as described above, i.e., an FR5 schedule of reinforcement was used. During an unavailability block the house light was off and responses on both levers were without scheduled consequences. Responding became more variable during the latter blocks in the session, likely as a result of satiety. We therefore used the amount of responses made during the first 5 min unavailability block as the critical parameter, because this block was flanked by two availability blocks in which animals always obtained the maximum amount of rewards within the time available. The animals received 10 sessions before the diet and 15 sessions after the diet. The mean number of responses during the first unavailability block of the last 4 sessions was used as the time-out score of the animal.

### Progressive ratio schedule of reinforcement

Under the progressive ratio schedule of reinforcement, the animals had to meet a response requirement on the active lever that progressively increased after every earned chocolate Ensure reward (1, 2, 4, 6, 9, 12, 15, 20, 25, etc [53]). The session started with illumination of the house light (signalling availability of the reward) and insertion of both the active and the inactive lever. Meeting the response requirement on the active lever resulted in retraction of both levers, illumination of the cue light above the active lever for 10 sec and delivery of 0.2 ml chocolate Ensure. After a 20 sec timeout, a new cycle started. The session ended when the animals failed to earn a reward within 60 min. Animals received 4 PR sessions before and 4 PR sessions after the diet. In both cases the average of the active lever responses over the 4 sessions was used as the PR score of the animal.

### Punished responding

The procedure was adapted from Deroche-Gamonet et al. (2004). During this procedure, the animals were tested in operant conditioning chambers that were different from those used during the training, time-out and PR sessions. The session started with illumination of the house light and presentation of both levers. During these sessions, animals responded under a FR5 schedule of reinforcement, in which each 1st lever press resulted in the presentation of a tone and each 4^th^ and 5^th^ lever press resulted in presentation of an electric foot shock (0.35mA, 2sec), administered via the grid floor. Each 5^th^ lever press resulted in delivery of 0.2 ml chocolate Ensure. The tone was turned off after the 4^th^ lever press or when the animals failed to make 4 responses within 1 minute, in which case a new FR5 cycle started. The outcome measure was the amount of lever presses that animals made during a session as a percentage of baseline responding (the average of 4 FR5 sessions the days before). We assessed responding under this paradigm in a pilot study (described above), in which electric footshock nearly completely suppressed responding for food in all animals.

### Quinine adulteration

Animals were given free access to either unadulterated or adulterated (using 2 mM quinine; Sigma, The Netherlands) chocolate Ensure in the home cage for 30 min on different days. A pilot experiment showed that a concentration of 2 mM quinine resulted in substantial individual variability, while higher concentrations suppressed intake in almost all animals, and lower concentrations had very little effect on chocolate Ensure intake. The suppression ratio was calculated as follows: ((un-adulterated consumption - adulterated consumption) / un-adulterated consumption) * 100, so that a suppression ratio of 100 comprised full suppression of intake, and a ratio of 0 meant no suppression at all.

### Reward devaluation

Animals were given 2 h of free access to chocolate Ensure in the home cage immediately before an operant session of 20 min, during which the house light was illuminated and both levers were present throughout the session. Both active and inactive lever responses were without scheduled consequences. The devaluation score was calculated as the amount of active lever presses made by the animal after devaluation. Results were compared to the amount of lever presses during a normal 20 min non-devalued FR5 session the day before.

### Extinction and reinstatement

Animals received 12 daily 1 h operant sessions during which lever presses were without scheduled consequences. The house light (that previously signalled reward availability) was turned on throughout the session. On day 13, cue-induced reinstatement was tested as follows. The session started with illumination of the cue light above the active lever for 10 sec. During this session, meeting the FR5 requirement on the active lever resulted in retraction of both levers and illumination of the cue light for 10 sec, but no reward was delivered. Animals received normal extinction sessions on day 14 and 15. On day 16, chocolate Ensure-induced reinstatement was tested. The session started with delivery of 0.6 ml of chocolate Ensure. Lever presses during this session were without scheduled consequences.

### Data analysis

Based on the three criteria, an ‘addiction score’ was calculated according to Belin et al. [54]. Normalization was done by subtracting the mean of all animals from every individual animal and dividing by the standard deviation of the whole group. This resulted in a criterion score with an average of 0 and a standard deviation of 1 for each criterion. The addiction score was then calculated as the sum of three normalized scores. We also categorized the animals according to Deroche–Gamonet et al., meaning that we counted the number of criteria for which the animal scored between the 66^th^ and 99^th^ percentile of the distribution [37]. The two diet groups were compared to each other using Student’s t-tests. The criteria groups were compared using one-way ANOVAs followed by Turkey’s multiple comparison post-hoc tests, where appropriate. Raw data sets are available upon request.

## Results

**Figure 1:**
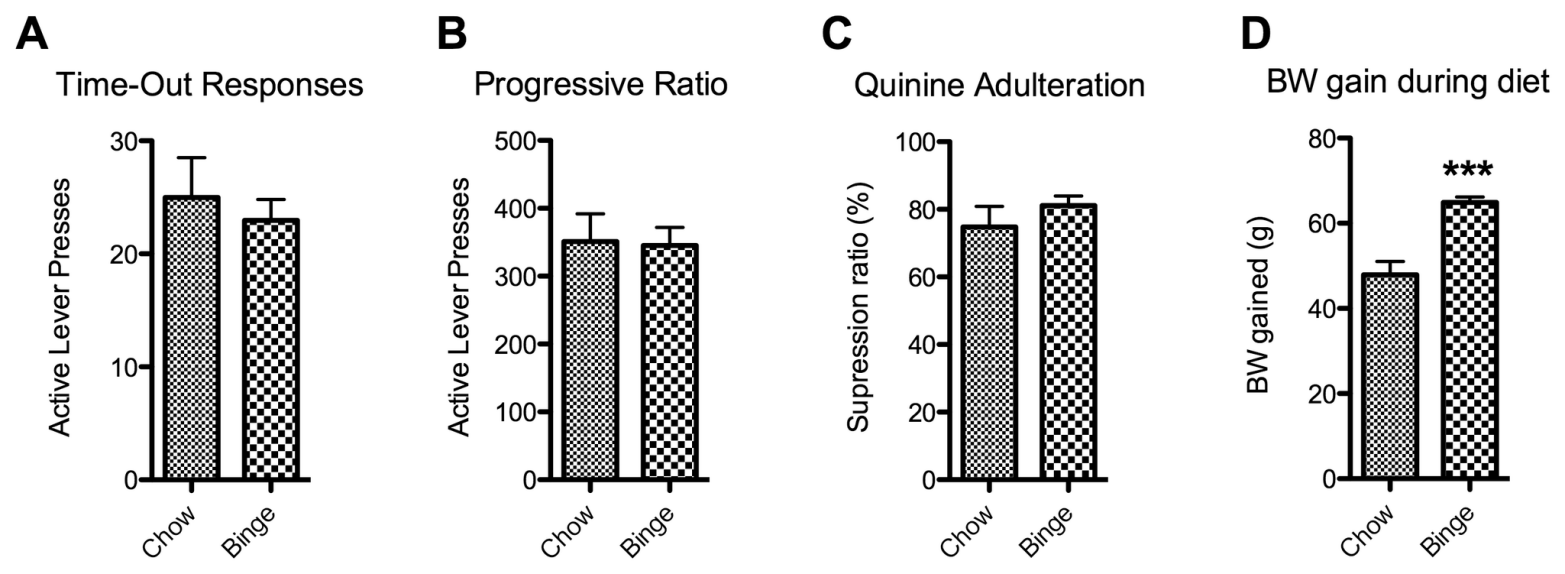
The effect of the binge diet on operant responding and body weight gain. Panel A and B show mean lever presses (+SEM) (y-axis) per diet group (x-axis) during the first time-out during a time-out paradigm (A) or while working under a progressive ratio schedule of reinforcement (B). Panel C shows the mean suppression ratio (+SEM) (y-axis) of chocolate consumption caused by adulteration with 2mM quinine. Panel D shows the mean increase in body weight in grams (+SEM) (y-axis) during the 8 weeks of the diet. *** Indicates significant difference between the groups (p<0.0001).

**Figure 2:**
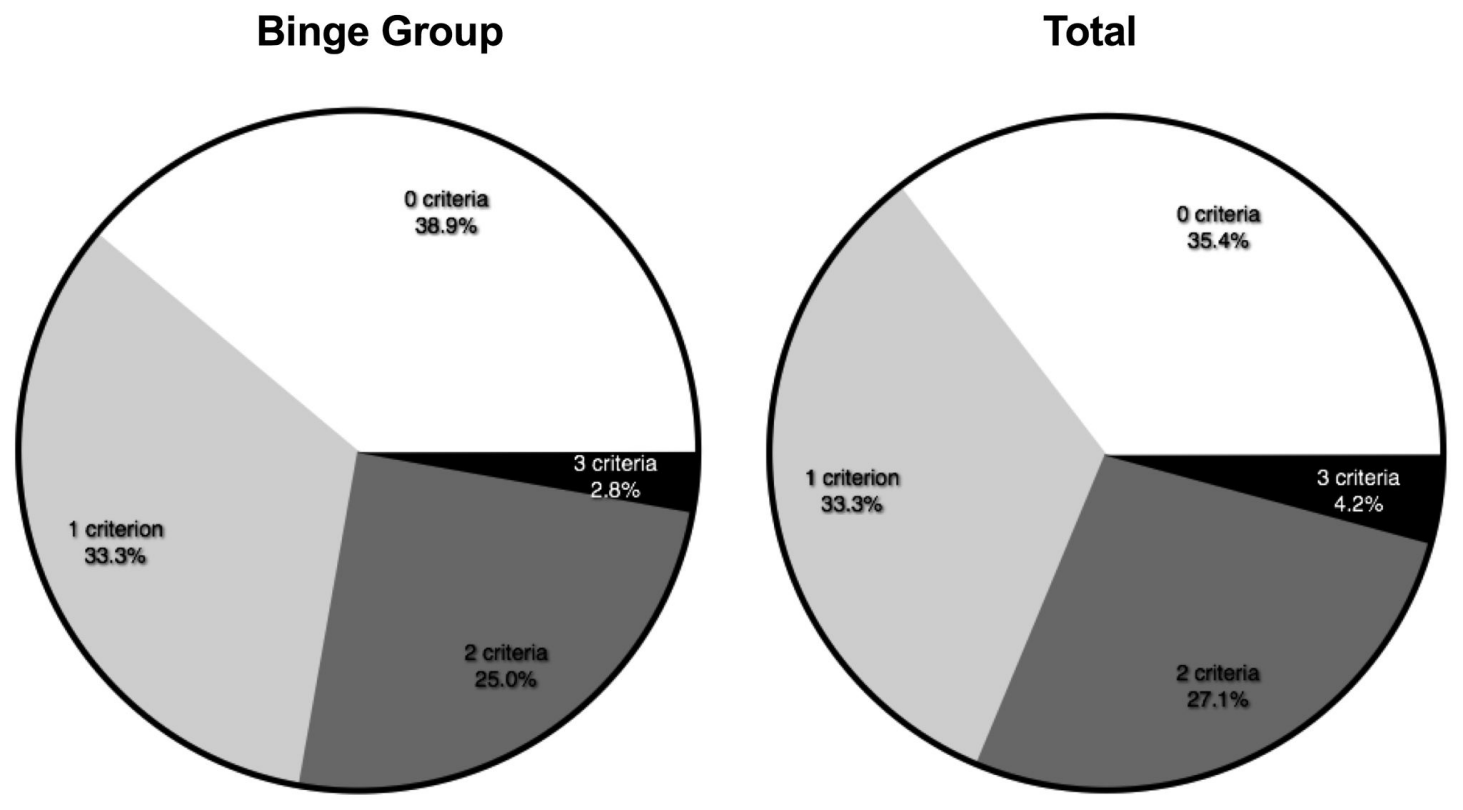
The distribution of the different criteria groups. Animals were assigned to a criteria subgroup based on the amount of criteria for which they scored between the 66^th^ and 99^th^ percentile. The left panel shows the distribution in the animals that were exposed to the binge diet, whereas the right panel shows the distribution throughout the whole cohort.

**Figure 3:**
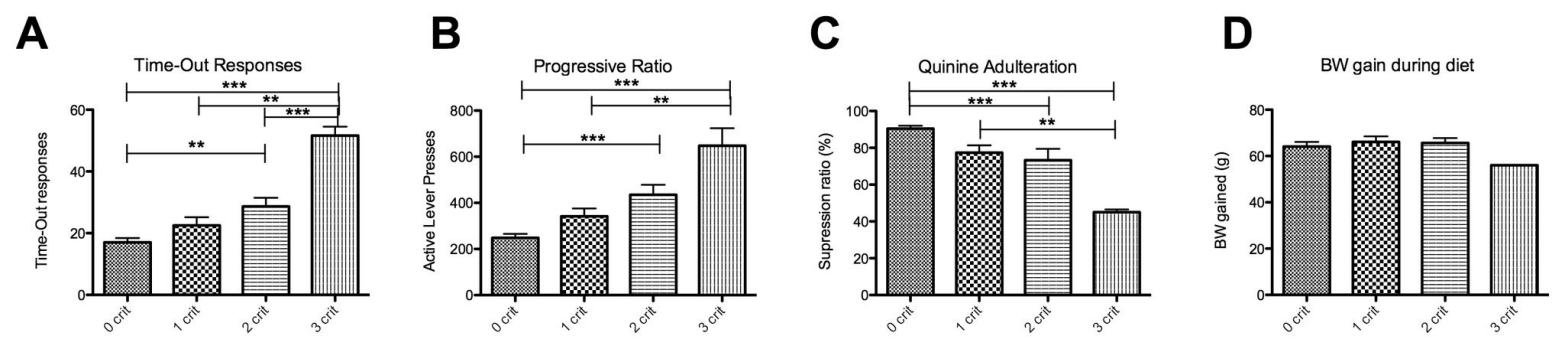
Differences in operant responding between the criteria subgroups. Panel A and B show mean operant responses (+SEM) (y-axis) per criteria subgroup (x-axis) either during the first time-out (A) or under a progressive ratio schedule of reinforcement (B). Panel C represents the mean suppression ratio (+SEM) (y-axis) by 2mM quinine adulteration per criteria subgroup (x-axis). Panel D depicts the mean body weight gain (+SEM) from the animals in the binge group during the diet. **: P<0.001, ***:P<0.0001.

A cohort of animals (n=48) was tested for the three criteria of addiction-like behavior. In order to provoke the development of uncontrolled eating, a subgroup (n=36) was exposed to a binge model. No significant differences on any of the three individual criteria between control and binge animals were observed (time out responding (TO): p=0.6 t=0.53 df = 46; progressive ratio (PR): p=0.9 t=0.1128 df = 46; quinine: p=0.3 t=1.048 df = 46) (Figure 1A–C). The binge model did, however, result in a significant increase in body weight gain (p<0.0001 t=6.105 df = 46) (Figure 1D). Next, we divided all animals into 4 subgroups based on the amount of criteria for which they scored between the 66^th^ and 99^th^ percentile, according to Deroche-Gamonet et al. (2004). In our case, the 3-critt subgroup was not larger than expected by chance (i.e., 3,6%) (Figure 2). This was true for both the binge group (Figure 2A) as well as the whole cohort (Figure 2B). The criteria subgroups differed from each other on each criterion (ANOVA TO: p<0.0001 F=11.42 df = 47; PR: p<0.0001 F=9,850 df = 47; quinine: p=0.0006 F=6.932 df = 47) (Figure 3A–C). In the binge group we assessed if decreased control predicted body weight gain during the diet, which was not the case (Figure 3D).

**Figure 4:**
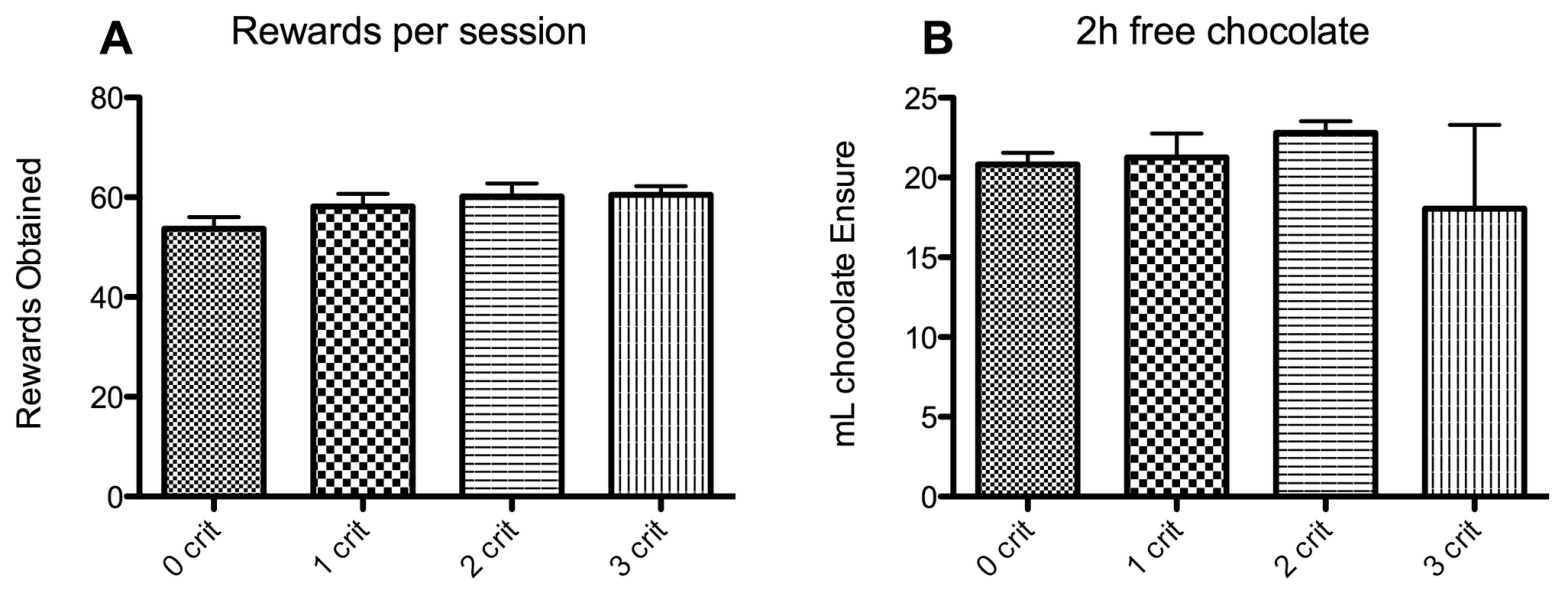
Chocolate consumption. Panel A shows the mean rewards obtained in a normal Time-Out session (+SEM) (y-axis). No difference is observed between criteria groups (x-axis). Panel B shows mean chocolate intake in ml (+SEM) (y-axis) during 2h of ad libitum access in the home cage. No difference was observed between the criteria groups (x-axis).

**Figure 5:**
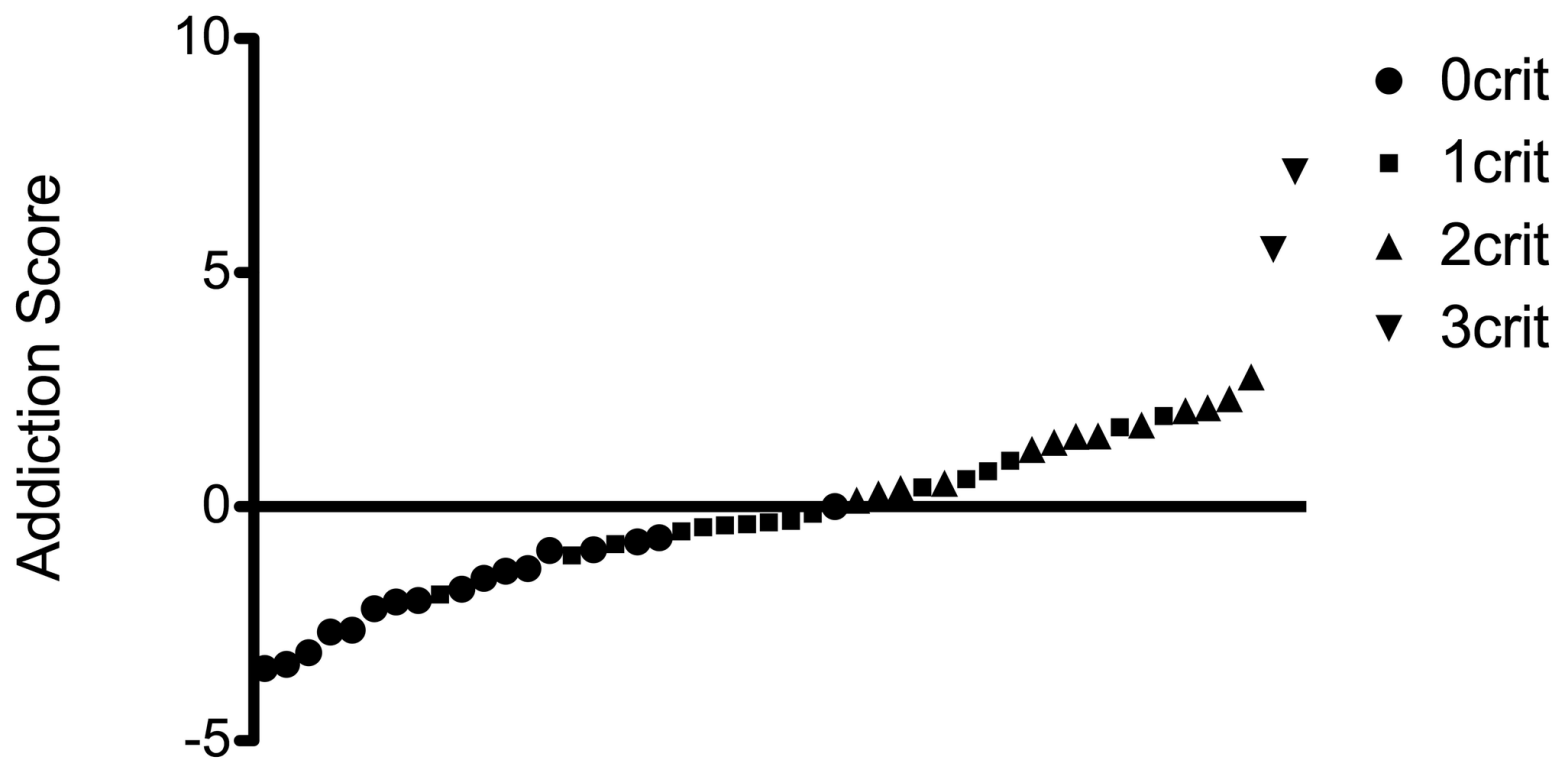
Range of addiction scores divided by criteria group. The addiction score (sum of the normalized scores for three criteria) is indicated on the y-axis. Animals are ranked from low to high addiction score and divided by criteria group as indicated by the symbols.

Importantly, the differences between the criteria groups were not caused by variation in satiety or energy requirement as all groups consumed the same amount of chocolate during a 70 min FR5 session (ANOVA p=0.3 F=1.266 df = 47) (Figure 4A) or when given 2h of ad libitum access to chocolate Ensure (ANOVA p=0.4 F=0.9651 df = 47) (Figure 4B). We also calculated the addiction score according to [54]. This resulted in a wide range of scores (Figure 5).

**Figure 6:**
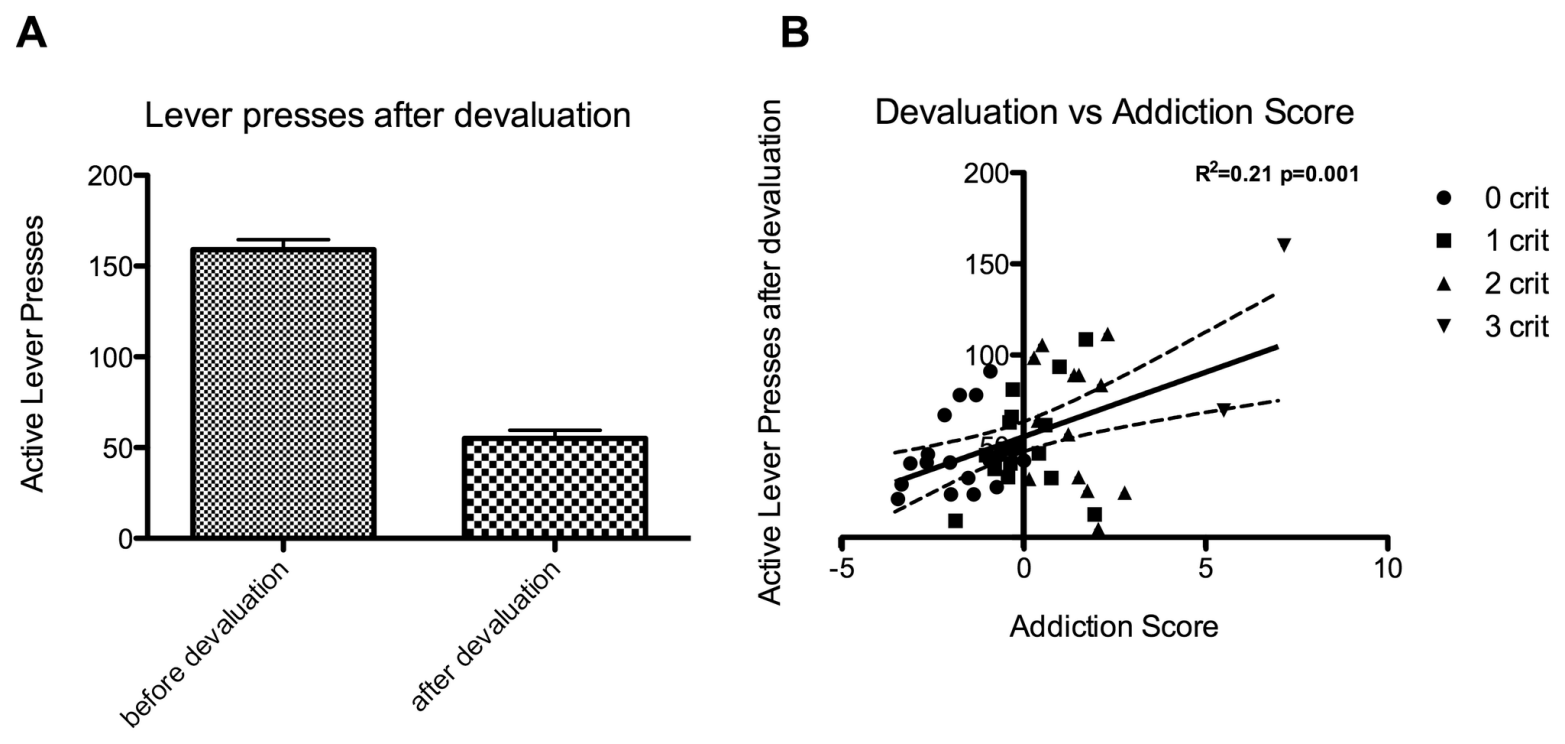
The effect of satiety-induced devaluation on responding in extinction. Panel A shows active lever presses made (+SEM) (y-axis) either during a 20min FR5 session (before devaluation) or during a 20min operant session in which lever presses where non-reinforced that was preceded by 2h of ad libitum access to chocolate ensure (after devaluation). Panel B shows the active lever presses made during the session after devaluation (y-axis) as a function of the addiction score (x-axis). A black line indicates the best fit of a linear regression analysis; dashed lines indicate the 95% confidence interval of the best fit.

It has been suggested that the formation of aberrant, drug-directed stimulus-response habits is a critical step in the development of addictive behavior [46,47]. To assess if the behavior expressed by the animals was goal-directed or habitual, we devalued the chocolate Ensure reward by giving the animals 2 h of free access in their home cage prior to a 20 min operant testing session during which lever presses where not reinforced. The animals made on average 63% less responses when the chocolate was devalued compared to a 20 min session in which lever presses where reinforced and the chocolate was not devalued (Mean difference is 104.0, 95%c.i.= 92.06 to 115.9) (Figure 6A). Lever presses made after devaluation correlated with addiction score (r^2^=0.2, p<0.001) (Figure 6B). No difference between binge and control group was observed (data not shown).

**Figure 7:**
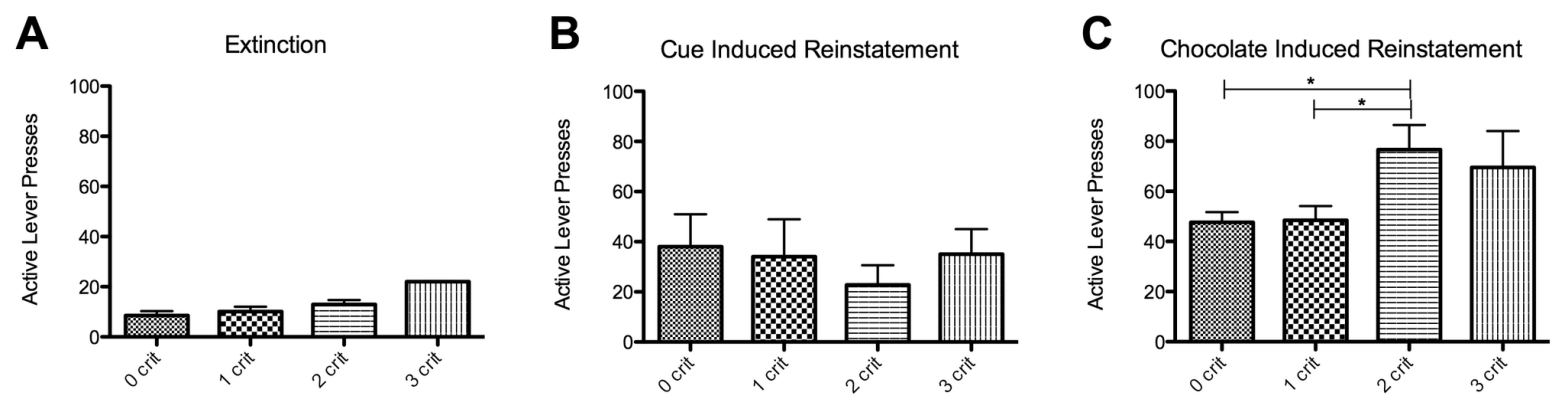
Propensity to reinstate per criteria group. This figure shows the mean lever presses (+SEM) (y-axis) made during the last 60min extinction session (panel A), cue-induced reinstatement session (panel B) or chocolate induced reinstatement session (panel C) divided per criteria group (x-axis). * Indicates significant difference between the groups (p<0.05).

Next, we assessed if animals with diminished control over eating were more prone to reinstate extinguished responding. We measured 2 types of reinstatement. As compared to responding during extinction (Figure 7A), response-contingent presentation of the chocolate Ensure-associated cues engendered significant (p=0.0035 t=3.077 df = 47) reinstatement of responding over the whole cohort, but there was no difference between the criteria groups (ANOVA p=0.865 F=0.2442 df = 47) (Figure 7B). During chocolate Ensure-induced reinstatement, we observed significant reinstatement (p<0.0001 t=12.35 df = 47) and a significant difference in reinstatement between groups, with the 2 criteria group showing higher levels of responding than the 0 and 1 criteria animals (ANOVA p=0.01 F=4.225 df = 47) (Figure 7C).

## Discussion

In the present study, we adapted an animal model of addiction-like behavior for cocaine to assess the occurrence of addictive behavior directed at palatable food. In order to facilitate the development of uncontrolled eating, a subgroup of the animals (n=36) was exposed to a binge-type model consisting of 4 days of 66% of ad libitum chow alternated with 3 days access to ad libitum chow in combination with Oreo Cookies. After testing for the three criteria of loss of control, we also measured responding after devaluation and the propensity to reinstate extinguished responding induced by response-contingent presentation of the food reward-associated cue or the chocolate Ensure reward itself.

### A binge model does not affect control over food seeking

We did not observe an effect of the binge model on any on the three criteria for addiction-like behavior (Figures 1 and 2). We did, however, observe an increase in body weight gain after exposure to the binge model. The current diet is based on a study by Hagan et al., who showed increased bingeing on palatable food of animals who had been exposed to a comparable diet even after they had been withdrawn from this diet for 30 days [50]. In contrast to Hagan et al., we used male rats. We can therefore not exclude that we might had obtained more pronounced effects of the binge diet had we used female rats. Indeed, BED is more prevalent in human females then in males [55]. On the other hand, it has been repeatedly shown that, given the right circumstances, both male and female rats will binge on palatable food [56–58]. Another commonly used binge model, that causes bingeing in both sexes of rats, uses alternating 12h/12h periods of food deprivation combined with access to a 10% sucrose solution [59,60]. Previous research has also shown that constant access to a high fat-high sucrose diet increases responding under a PR schedule and responding under a PR schedule before access to the diet positively correlates with abdominal fat storage after 4weeks of access to a high-fat high-sugar diet in male rats [51]. Thus, exposure to certain types of obesogenic diets can lead to bingeing and increased motivation for food. However, our data indicate that prolonged exposure to a binge diet is in itself not sufficient to evoke clear-cut addiction-like behavior.

### No evidence for ‘food-addiction’, but high individual variability in control over palatable food intake

Contrary to what has been found for cocaine, the subgroup of rats that performed in the upper tertile for all three criteria was not larger than expected by chance (3,6%). Therefore, it is reasonable to conclude that no clear-cut signs of addiction-like behavior directed at chocolate Ensure developed in our study. Even in the absence of such an ‘addicted-subgroup’, the range of control over food seeking observed in the present study is highly relevant. That is, diminished control over food intake in humans, even in the absence of clear addiction-like behavior, may cause overeating and prolonged mild overeating leads to obesity in some individuals. In the present study, decreased control over palatable food intake did not predict body weight gain, which is likely due to the fact that rats (in contrast to humans) do not try to prevent body weight gain. Thus, the neural mechanisms behind this continuum of control over food seeking and taking are important to investigate and our current model provides the behavioral tools to do so.

### Animals showing diminished control over food intake are less sensitive to reward devaluation

We observed a significant decrease in responding after devaluation on a group level (Figure 6a). Interestingly, there were large individual differences regarding the impact of devaluation, which correlated with the addiction score (Figure 6b). It has been proposed that the development of addiction is facilitated by a switch from goal directed outcome-driven behavior towards habitual stimulus-driven behavior [46]. The former is thought to be mediated by ventral and medial parts of the striatum, whereas the latter is depends on the dorsolateral striatum [61]. Indeed, it has been repeatedly shown that prolonged cocaine self-administration recruits dorsolateral striatal mechanisms underlying drug seeking [62–65] and that lesions or inactivation of the dorsolateral striatum reduces habitual behavior [66–69]. Since animals that show less control over food intake express more habitual behavior, these findings suggest that reduced control over food intake is associated with a greater dorsolateral striatal involvement in the control over eating.

### Low control animals are more prone to reinstate extinguished food-seeking

A prominent feature of addiction is the high risk of relapse [70,71]. This can be investigated using animal models that study the propensity of an animal to reinstate drug seeking following extinction of the operant response. Drug seeking can be reinstated using a drug-associated cue, a small ‘priming’ amount of the drug or by stress [38]. To assess if animals with less control over their food seeking were more likely to reinstate extinguished food-seeking, we tested the animals for both cue- and reward-induced reinstatement. As seen in Figure 7C, only priming the animals with the chocolate-flavored reward induced significant difference in reinstatement between the 4 criteria groups. In this case 2 criteria animals responded significantly more during reinstatement. It is likely that the 3 criteria animals are also more likely to reinstate, but this was difficult to demonstrate statistically because of the small number of animals in this group.

In conclusion, we present a model that can be used to measure changes in the control over eating behavior. The model produces a continuum of behavior ranging from very high to low control, the extreme of which might be termed food addiction, but at least in the current experiment, no clear boundary between ‘addicted’ and ‘non-addicted’ animals can be drawn, nor is the subgroup of animals that can potentially be classified as showing addiction-like behavior greater than expected by chance. On the other hand, we found that low control over food intake was associated with a high propensity of palatable food-induced relapse and increased habitual responding for chocolate, indicating that behavioral changes associated with addictive behavior can be seen in animals with low control over palatable food intake. The model therefore provides a valuable tool to study control over eating and its neural underpinnings. This is highly relevant when we consider that diminished control over eating, even without the strict classification of food-addiction, may result in severe health problems.
